# Resident Involvement in Hip Arthroscopy Procedures Does Not Affect Short-Term Surgical Outcomes

**DOI:** 10.1016/j.asmr.2021.06.005

**Published:** 2021-08-19

**Authors:** Connor R. Crutchfield, Jack R. Zhong, Nathan J. Lee, David P. Trofa, T. Sean Lynch

**Affiliations:** Columbia University Irving Medical Center, New York, New York, U.S.A.

## Abstract

**Purpose:**

To evaluate whether the presence of residents in hip arthroscopy (HA) procedures affects short-term surgical outcomes.

**Methods:**

The American College of Surgeons National Surgical Quality Improvement Program Database was used to identify patients who underwent HA from 2006 to 2012. Demographic and 30-day outcome variables were compared between cohorts of patients with and without residents. Multivariate logistic regression was used to identify whether resident involvement was an independent risk factor for adverse outcomes. Propensity score matching was performed to control for all demographic and intraoperative variables.

**Results:**

A total of 869 patients (59.7% female) were included in this study, 626 of which reported data on resident involvement. Patients were mostly White (73.4% of cases without a resident, 51.8% with a resident, *P* < .05). Those with residents were younger (*P* = .016), had lower modified 5-item frailty index (mFI-5) scores (*P* = .028), and had fewer cardiac comorbidities (*P* = .008). There was no difference in diabetic status, dyspnea symptoms, history of chronic obstructive pulmonary disease, renal comorbidity, neurologic comorbidity, cumulative comorbidities, history of bleeding disorders, inpatient vs. outpatient treatment, preoperative functional status, smoking history, and steroid use for chronic conditions. There was no difference in all complications, operative time, length of stay, reoperation, readmission, wound complication, venous thromboembolism, blood transfusions, or sepsis. Propensity score match for demographic and intraoperative differences found no association between resident involvement and increased complications. Resident involvement was not an independent risk factor for all complications studied.

**Conclusion:**

Resident involvement in HA procedures was not a risk factor for 30-day complications between 2006 and 2012. Resident involvement did not increase the risk of adverse outcomes, readmission, reoperation, or length of stay, nor did it significantly increase operative times.

## Introduction

Participative surgical experience is the mainstay of a residency in orthopedic surgery, where at least 455 procedures in a range of orthopedic subspecialties must be completed before graduation.^1^ Teaching hospitals have long involved residents in the operating room (OR), using an apprenticeship model to teach technical skills and surgical judgment that will prepare them to practice independently. However, factors like the evolving legal milieu in medicine, the focus of the current health care reform on patient-driven outcomes and value-based care, and work hour restrictions^2^ have brought resident participation in the OR under scrutiny and impacted graduate medical training as a whole.^3^, ^4^, ^5^, ^6^, ^7^ Given half of all surgical procedures in the United States are performed in teaching hospitals^8^ and that resident involvement is weighted toward the latter stages of patient care (perioperative, intraoperative, and postoperative care),^9^ the education of surgical residents is integral to the patient experience and must be optimized to ensure quality surgical outcomes today and competent surgeons for the future.

In light of this, there have been studies published that examine the surgical outcomes of procedures involving resident participation and the existence of a possible July phenomenon.^10^, ^11^, ^12^ Current opinion is conflicted as to whether or not resident involvement is a detriment to surgical outcomes, and results vary by specialty. Some studies have demonstrated no significant differences in outcomes with versus without residents,^13^, ^14^, ^15^, ^16^, ^17^, ^18^, ^19^, ^20^, ^21^, ^22^ while others have argued for the protective effects of resident involvement,^13^^,^^19^^,^^23^, ^24^, ^25^, ^26^ and still a third group has found that resident presence is accompanied by an increase in morbidity and/or mortality.^8^^,^^24^^,^^27^, ^28^, ^29^ Even within studies, there is conflicting overlap where, for example, morbidity rates increase, but mortality rates decrease.^24^ Regardless of outcome though, much of the existing literature has found that resident involvement in surgery increases operative times.^14^^,^^23^^,^^25^^,^^28^^,^^30^, ^31^, ^32^ Prolonged surgeries on account of resident education do raise concern about the increased risk of complications,^24^^,^^33^ but the foregoing studies have also shown this is not necessarily the case. In fact, Kazaure et al.^16^ demonstrated in 2012 that attending surgeons exercise sound judgement in how they educate their residents so as to not jeopardize patient outcomes.

In a myriad of orthopedic studies, resident involvement was not linked to an increase in morbidity or mortality.^13^^,^^17^^,^^18^^,^^20^, ^21^, ^22^^,^^26^ These findings are especially promising, since it is thought that residents are more likely to be involved in more rare and complex “teaching cases”.^9^^,^^24^ Hip arthroscopy (HA) is a relatively recent procedure that has seen an explosion in case volume in recent years,^34^ yet there are currently few studies examining resident participation in hip cases and no studies focusing on HA specifically. Given the specific nature and complexity of HA for junior surgeons, the presence of residents in HA is of particular interest because it may disproportionately affect surgical outcomes in comparison to other orthopedic specialties. The purpose of this study is to evaluate whether the presence of residents in hip arthroscopy procedures affects short-term surgical outcomes. We hypothesized that resident involvement would significantly impact operative time but not postoperative complication rates.

## Methods

The American College of Surgeons National Surgical Quality Improvement Program database (ACS NSQIP) is a deidentified database with high-quality information on procedure type and complication data from more than 680 hospitals across the United States.^35^^,^^36^ In February of 2020, the ACS NSQIP was retrospectively queried for hip arthroscopy cases that involved resident participation between 2006 and 2012—the last year that data on resident involvement was collected.^17^ Current Procedural Terminology (CPT) codes 29860, 29861, 29862, 29863, 29914, 29915, 29916, and 29999 were used to isolate and identify hip arthroscopy procedures (Table 1). Cases with the CPT code 29999, referring to an unspecified musculoskeletal arthroscopy, were included if they were associated with other *International Classification of Diseases, Ninth Revision* (ICD-9) billing codes, indicating hip pathology. Other information collected included patient demographics, medical comorbidities, intraoperative details, operative time, and postoperative complications up to 30 days. Cases that did not report the presence of a resident versus attending physician were excluded from analysis. This study was performed at the Columbia University Irving Medical Center (New York, NY).

**Table 1:** List of Hip-Specific CPT and ICD-9 Codes Used for NSQIP Query

CPT Code	Description
29860	Hip arthroscopy, diagnostic with or without biopsy
29861	Hip arthroscopy, removal of loose or foreign bodies/fragments; (e.g., chondral fragmentation)
29862	Hip arthroscopy, debridement, chondroplasty, abrasion arthroplasty, and/or resection of labrum (cleaning out inflammation or frayed labral/chondral tissue)
29863	Hip arthroscopy, synovectomy (e.g., plica resection or capsular plication)
29914	Hip arthroscopy; femoroplasty, shaving cam lesion off the femoral head/neck junction (includes chondroplasty where necessary)
29915	Hip arthroscopy; acetabuloplasty, shaving pincer lesion off the acetabular rim
29916	Hip arthroscopy, labral repair
29999	Hip arthroscopy, unlisted (includes concurrent procedures like removal of heterotopic bone, lysis of adhesions, or acetabular microfracture) Hip-related ICD-9 Codes: 715.15, 715.35, 715.95, 716.95, 718.05, 718.35, 718.65, 718.85, 718.95, 719.45, 719.65, 719.85, 719.95, 726.5, 733.42, 736.39, 754.32, 755.63, 843, and 843.8

CPT, current procedural terminology; ICD,  International Statistical Classification of Diseases and Related Health Problems.

### Variables Collected

The demographic information retrieved from the ACS NSQIP included sex, age, inpatient/outpatient status, and race. Perioperative comorbidity variables like diabetic status, dyspnea, history of severe chronic obstructive pulmonary disease, bleeding disorders, American Society of Anesthesiologist class (ASA) (3 vs ≥3), steroid use for a chronic condition, recent smoking history (within one year), and functional health status (independent vs dependent) were also collected. Obesity was calculated from patients’ heights and weights, and renal, neurological, cardiac, and cumulative comorbidities were also recorded. The outcomes of interest included operative time, postoperative length of stay (≤1 days vs >1 day), surgical complications, wound complications, venous thromboembolism (DVT), urinary tract infection (UTI), blood transfusions, sepsis, reoperations, and readmissions within 30 days of surgery. From these data, the 5-Factor Modified Frailty Index (mFl-5) of each patient was calculated as well. A higher mFl-5 score is associated with increased postoperative morbidity.^37^

### Statistical Analysis

The sample was stratified into two cohorts based on the presence or absence of a surgical resident scrubbed into the case. Comparisons of demographics, comorbidities, and outcome variables were made between the cohorts using χ^2^ tests for categorical variables and Kruskal-Wallis tests for the continuous variables of age and mFl-5 values. Multiple stepwise logistic regression was then used to identify whether resident involvement was an independent risk factor for adverse outcomes with a 95% confidence interval. A 1:1 propensity score algorithm was used, as previously described,^17^^,^^25^^,^^26^ to match race (White vs. non-White), cardiac comorbidities, age, and mFl-5 scores between resident and no resident cohorts in order to adjust for any confounding variables that could influence resident assignment to a case based on patient characteristics. Finally, bivariate analysis of surgical outcomes was conducted against resident involvement in the matched cohorts. All statistical analyses were conducted using SAS 9.2 software (SAS Institute, Inc., Cary, NC), and the α was set at *P* <.05 to define significance.

## Results

Querying the ACS NSQIP database identified 2,421 patients who underwent HA arthroscopy between 2006 and 2012. After applying preliminary inclusion criteria, a total of 869 patients remained. Two hundred forty-three (28.0%) patients were removed because of missing information regarding resident involvement, and the remaining 626 patients (59.7% female, 35.8% resident involvement) were included for analysis.

### Unmatched Analysis

In the unmatched cohorts, the mean patient age was 40.2 ± 14.0 years, and the mean mFl-5 score was 0.04 ± 0.97. There were 374 (59.7%) female patients and 166 (26.5%) obese patients. Patient age, mFl-5 score, race, and cardiac comorbidity were found to vary significantly by resident involvement, wherein resident cases involved patients that were younger, had lower mFl-5 scores, were less likely to be White, and less likely to have a cardiac comorbidity (*P* < .05 for all). No other patient demographic variables were found to vary significantly between cohorts (Table 2).

**Table 2:** Hip Arthroscopy Demographics Bivariate Analysis by Resident Involvement

Variable	Missing (*N* = 243)	No Resident (*N* = 402)	Resident (*N* = 224)	Total (*N* = 869)	*P* Value
Sex					.842^1^
Female	161 (66.3%)	239 (59.5%)	135 (60.3%)	374 (59.7%)	
Male	82 (33.7%)	163 (40.5%)	89 (39.7%)	252 (40.3%)	
Age					**.016**^2^
*N*	243	402	224	626	
Mean (SD)	42.5 (14.4)	41.3 (14.7)	38.3 (12.5)	40.2 (14.0)	
Median	42.0	42.0	38.0	40.0	
Q1, Q3	31.0, 52.0	30.0, 50.0	29.5, 46.0	30.0, 48.0	
mFI-5 Index					**.028**^2^
*N*	243	402	224	626	
Mean (SD)	.0 (.1)	.044 (.097)	.029 (0.091)	.04 (.97)	
Median	.0	.0	.0	.0	
Q1, Q3	0.0, 0.0	0.0, 0.0	0.0, 0.0	0.0, 0.0	
Race					**.042**^1^
Missing	22 (9.1%)	75 (18.7%)	88 (39.3%)	163 (26.0%)	
American Indian or Alaska Native	1 (.4%)	1 (.3%)	1 (.50%)	2 (.3%)	
Asian	10 (4.1%)	4 (1.0%)	1 (.5%)	5 (.8%)	
Black or African American	7 (2.9%)	13 (3.2%)	15 (6.7%)	28 (4.5%)	
Hispanic	11 (4.5%)	14 (3.5%)	3 (1.3%)	17 (2.7%)	
White	191 (78.6%)	295 (73.4%)	116 (51.8%)	411 (65.7%)	
Obese					.385^1^
No	168 (69.1%)	300 (74.6%)	160 (71.4%)	460 (73.5%)	
Yes	75 (30.9%)	102 (25.4%)	64 (28.6%)	166 (26.5%)	
ASA					.121^1^
<3	213 (87.7%)	358 (89.1%)	208 (92.9%)	566 (90.4%)	
≥3	30 (12.3%)	44 (10.9%)	16 (7.1%)	60 (9.6%)	
Diabetes					.786^1^
No	233 (95.6%)	384 (95.5%)	215 (96.0%)	599 (95.7%)	
Yes	10 (4.1%)	18 (4.5%)	9 (4.0%)	27 (4.3%)	
Dyspnea					.877^1^
Yes at Moderate Exertion	3 (1.2%)	6 (1.5%)	3 (1.34%)	9 (1.4%)	
No	240 (98.8%)	396 (98.5%)	221 (98.7%)	617 (98.6%)	
History of severe COPD					.929^1^
No	239 (98.4%)	400 (99.5%)	223 (99.6%)	623 (99.5%)	
Yes	4 (1.6%)	2 (.5%)	1 (.5%)	3 (.5%)	
Cardiac comorbidity					**.008**^1^
No	204 (84.0%)	333 (82.8%)	203 (90.6%)	536 (85.6%)	
Yes	39 (16.0%)	69 (17.2%)	21 (9.4%)	90 (14.4%)	
Neurological comorbidity					0.843^1^
No	243 (100.0%)	399 (99.3%)	222 (99.1%)	621 (99.2%)	
Yes	0 (.0%)	3 (.8%)	2 (.9%)	5 (.8%)	
Renal comorbidity					NA
No	243 (100.0%)	402 (100.0%)	224 (100.0%)	626 (100.0%)	
Cumulative comorbidities					.919^1^
No	145 (59.7%)	255 (63.6%)	143 (63.8%)	398 (63.6%)	
Yes	98 (40.3%)	147 (36.4%)	81 (36.2%)	228 (36.4%)	
History of bleeding disorders					0.326^1^
No	240 (98.8%)	397 (98.8%)	223 (99.6%)	620 (99.0%)	
Yes	3 (1.2%)	5 (1.2%)	1 (.5%)	6 (1.0%)	
In-patient/out-patient					.328^1^
In-patient	20 (8.2%)	32 (8.0%)	23 (10.3%)	55 (8.8%)	
Out-patient	223 (91.8%)	370 (92.0%)	201 (89.7%)	571 (91.2%)	
Functional health status prior to surgery					.750^1^
Independent	243 (100.0%)	397 (98.8%)	222 (99.1%)	619 (98.9%)	
Partially dependent	0 (.0%)	4 (1.0%)	2 (.90%)	6 (1.0%)	
Totally dependent	0 (.0%)	1 (.3%)	0 (.0%)	1 (.2%)	
Current Smoker Within One Year					.309^1^
No	199 (81.9%)	331 (82.3%)	177 (79.0%)	508 (81.2%)	
Yes	44 (18.1%)	71 (17.7%)	47 (21.0%)	118 (18.8%)	
Steroid Use for Chronic Condition					.113^1^
No	238 (97.9%)	400 (99.5%)	220 (98.2%)	620 (99.0%)	
Yes	5 (2.1%)	2 (.5%)	4 (1.8%)	6 (1.0%)	

NOTE. Boldface indicates statistical significance (*P* < .05).

ASA, American Society of Anesthesiologist class; COPD, chronic obstructive pulmonary disease; mFl-5, 5-factor modified frailty index.

1 Chi-Square.

2 Kruskal-Wallis.

There were a total of 18 (2.9%) complications with a mean 30-day reoperation rate of 0.8%, a readmission rate of 0.8%, and an overall mean operative time of 99.9 ± 54.5 minutes. The operative time of the resident cohort was 105.6 ± 59.4 and that of the no resident cohort was 96.6 ± 51.3. Patients left the hospital the same day of surgery 94.6% (*n* = 592) of the time and only 1.1% (*n* = 7) experienced wound complications. No significant differences were found between any of the collected outcome variables; however, longer operative times and a lower complication rate in cases involving residents trended toward significance (*P* = 0.069 and *P* = 0.076, respectively; Table 3). Logistic regression analysis indicated that resident involvement was not an independent predictor of surgical complications, readmissions, reoperations, wound complications, DVT, sepsis, blood transfusions, operative time, and length of stay (Table 4).

**Table 3:** Hip Arthroscopy Morbidity Bivariate Analysis by Resident Involvement.

Variable	Missing (*N* = 243)	No Resident (*N* = 402)	Resident (*N* = 224)	Total (*N* = 869)	*P* Value
Any complication					.076^1^
No	228 (93.8%)	394 (98.0%)	214 (95.5%)	608 (97.1%)	
Yes	15 (6.2%)	8 (2.0%)	10 (4.5%)	18 (2.9%)	
Total operation time (min)					.069^2^
*N*	243	402	224	626	
Mean (SD)	115.7 (67.7)	96.6 (51.3)	105.6 (59.4)	99.9 (54.5)	
Median	99.0	83.5	91.0	86.0	
Q1, Q3	75.0, 145.0	59.0, 120.0	65.5, 140.5	61.0, 126.0	
Length of stay					.759^1^
≤1 days	226 (93.0%)	381 (94.8%)	211 (94.2%)	592 (94.6%)	
>1 days	17 (7.0%)	21 (5.2%)	13 (5.8%)	34 (5.4%)	
Reoperation in 30 days					.257^1^
No	243 (100.0%)	400 (99.5%)	221 (98.7%)	621 (99.2%)	
Yes	0 (.0%)	2 (.5%)	3 (1.3%)	5 (.8%)	
Readmission in 30 days					.257^1^
No	239 (98.4%)	400 (99.5%)	221 (98.7%)	621 (99.2%)	
Yes	4 (1.6%)	2 (.5%)	3 (1.3%)	5 (.8%)	
Wound complication					.233^1^
Missing	2 (.8%)	0 (.0%)	1 (.4%)	1 (.2%)	
No	236 (97.1%)	399 (99.3%)	219 (97.8%)	618 (98.7%)	
Yes	5 (2.1%)	3 (.8%)	4 (1.8%)	7 (1.1%)	
Venous thromboembolism					.455^1^
No	242 (99.6%)	401 (99.8%)	224 (100.0%)	625 (99.8%)	
Yes	1 (.4%)	1 (.3%)	0 (.0%)	1 (.2%)	
Urinary Tract Infection					NA
No	243 (100.0%)	402 (100.0%)	224 (100.0%)	626 (100.0%)	
Blood Transfusions					.465^1^
No complication	235 (96.7%)	399 (99.3%)	221 (98.7%)	620 (99.0%)	
Transfusions/intra-op/post-op	8 (3.3%)	3 (.8%)	3 (1.3%)	6 (1.0%)	
Sepsis					0.290^1^
No	241 (99.2%)	400 (99.5%)	224 (100.0%)	624 (99.7%)	
Yes	2 (.8%)	2 (.5%)	0 (.0%)	2 (.3%)	

1 Chi-Square.

2 Kruskal-Wallis.

**Table 4:** Stepwise Logistic Regression for Resident Involvement on 30-day Outcomes After Hip Arthroscopy

Outcome	Odds Ratio (95% Confidence Interval)	*P* Value
Any complication	1.787 (.426, 7.508)	.428
Readmission	5.123 (.53-49.556)	.158
Reoperation	2.396 (.187, 30.719)	.502
Wound complication	4.033 (.500, 32.546)	.255
Venous thromboembolism	<.001 (<.001, >999.999)	.949
Sepsis	.000 (.000,7.13E89)	.934
Intra-op/post-op transfusion	2.55 (.362, 17.964)	.347
Operative time >1.5 hours	<.001 (<.001, >999.999)	1.000
Length of stay >1 day	1.171 (.567, 2.419)	.671

### Matched Analysis

After matching patients, the resident and no resident cohorts each contained 224 patients with a mean patient age of 38.5 ± 13.2 years and a mean mFl-5 score of 0.03 ± 0.09. There were 269 female patients (60%), and 116 (25.9%) patients were obese. The overall mean complication rate was 3.3% with a 1.1% reoperation rate and a 0.9% readmission rate. The mean operative time was 104.7 ± 57.6, 4.5%. There were 10 (4.5%) complications in the resident cohort and 5 (2.2%) in the no resident cohort with mean operative times of 105.6 ± 59.4 minutes and 103.7 ± 55.8 minutes, respectively. Patients left the hospital the same day of surgery 94% (*n* = 421) of the time and only 1.1% (*n* = 5) experienced wound complications. Overall, the only demographic variable to vary by cohort was steroid use for a chronic condition (*P* = .045), with higher use among the patients in the resident cohort than the no resident cohort. There were no significant differences in the preoperative variables of obesity, ASA, diabetes, dyspnea, history of COPD, systemic comorbidities, bleeding disorders, inpatient/outpatient status, functional health status, or recent smoking history (Table 5). Similarly, no differences were found between cohorts in any of the 30-day outcome variables, including overall complications, operative time, length of stay, reoperation, readmission, wound complications, DVT, UTI, blood transfusions, or sepsis. A breakdown of all matched surgical morbidity outcomes is listed in Table 6.

**Table 5:** Hip Arthroscopy Demographics Bivariate Analysis by Resident Involvement—Propensity Score Matched

Variable	No Resident (*N* = 224)	Resident (*N* = 224)	Total (*N* = 448)	*P* Value
Sex				.923^1^
Female	134 (59.8%)	135 (60.3%)	269 (60.0%)	
Male	90 (40.2%)	89 (39.7%)	179 (40.0%)	
Age				.876^2^
*N*	224	224	448	
Mean (SD)	38.6 (13.9)	38.3 (12.5)	38.5 (13.2)	
Median	38.0	38.0	38.0	
Q1, Q3	27.0, 48.0	29.5, 46.0	28.0, 46.0	
mFI-5 Index				.416^2^
*N*	224	224	448	
Mean (SD)	.033 (.084)	.029 (.091)	.031 (.087)	
Median	.0	.0	.0	
Q1, Q3	.0, .0	.0, .0	.0, .0	
Race				.075^1^
Missing	72 (32.1%)	88 (39.3%)	160 (35.7%)	
American Indian or Alaska Native	1 (.4%)	1 (.4%)	2 (.4%)	
Asian	4 (1.8%)	1 (.5%)	5 (1.1%)	
Black or African American	12 (5.4%)	15 (6.7%)	27 (6.0%)	
Hispanic	14 (6.3%)	3 (1.3%)	17 (3.8%)	
White	121 (54.0%)	116 (51.8%)	237 (52.9%)	
Obese				.196^1^
No	172 (76.8%)	160 (71.4%)	332 (74.1%)	
Yes	52 (23.2%)	64 (28.6%)	116 (25.9%)	
ASA				.857^1^
<3	207 (92.4%)	208 (92.9%)	415 (92.6%)	
≥3	17 (7.6%)	16 (7.1%)	33 (7.4%)	
Diabetes				.815^1^
No	214 (95.5%)	215 (96.0%)	429 (95.8%)	
Yes	10 (4.5%)	9 (4.0%)	19 (4.2%)	
Dyspnea				1.000^1^
Yes at Moderate Exertion	3 (1.3%)	3 (1.3%)	6 (1.3%)	
No	221 (98.7%)	221 (98.7%)	442 (98.7%)	
History of severe COPD				.562^1^
No	222 (99.1%)	223 (99.6%)	445 (99.3%)	
Yes	2 (.9%)	1 (.45%)	3 (.7%)	
Cardiac comorbidity				.751^1^
No	201 (89.7%)	203 (90.6%)	404 (90.2%)	
Yes	23 (10.3%)	21 (9.4%)	44 (9.8%)	
Neurological comorbidity				.156^1^
No	224 (100.0%)	222 (99.1%)	446 (99.6%)	
Yes	0 (.0%)	2 (.9%)	2 (.5%)	
Renal comorbidity				
No	224 (100.0%)	224 (100.0%)	448 (100.0%)	NA
Cumulative comorbidities				.426^1^
No	151 (67.4%)	143 (63.8%)	294 (65.6%)	
Yes	73 (32.6%)	81 (36.2%)	154 (34.4%)	
Bleeding disorders history				.315^1^
No	221 (98.7%)	223 (99.6%)	444 (99.1%)	
Yes	3 (1.3%)	1 (.5%)	4 (.9%)	
In-patient/Out-patient				.630^1^
In-patient	20 (8.9%)	23 (10.3%)	43 (9.6%)	
Out-patient	204 (91.1%)	201 (89.7%)	405 (90.4%)	
Functional Health Status Prior to Surgery				.653^1^
Independent	221 (98.7%)	222 (99.1%)	443 (98.9%)	
Partially Dependent	3 (1.3%)	2 (0.9%)	5 (1.1%)	
Current Smoker Within One Year				.278^1^
No	186 (83.0%)	177 (79.0%)	363 (81.0%)	
Yes	38 (17.0%)	47 (21.0%)	85 (19.0%)	
Steroid Use for Chronic Condition				**.045**^1^
No	224 (100.0%)	220 (98.2%)	444 (99.1%)	
Yes	0 (.0%)	4 (1.8%)	4 (.9%)	

NOTE. Boldface indicates statistical significance (*P* < .05).

ASA, American Society of Anesthesiologist class; mFl-5, 5-Factor Modified Frailty Index; COPD, chronic obstructive pulmonary disease.

∗Propensity score match for age, mFI-5, cardiac comorbidity, and “White” race.

1 Chi-Square.

2 Kruskal-Wallis.

**Table 6:** Hip Arthroscopy Morbidity Bivariate Analysis by Resident Involvement—Propensity Score Matched

Outcome	No Resident (*N* = 224)	Resident (*N* = 224)	Total (*N* = 448)	*P-*value
Any complication				.189^1^
No	219 (97.8%)	214 (95.5%)	433 (96.7%)	
Yes	5 (2.2%)	10 (4.5%)	15 (3.3%)	
Total operation time (min)				.785^2^
N	224	224	448	
Mean (SD)	103.7 (55.8)	105.6 (59.4)	104.7 (57.6)	
Median	90.5	91.0	90.5	
Q1, Q3	61.5, 127.5	65.5, 140.5	63.5, 132.0	
Length of stay				.843^1^
≤1 days	210 (93.8%)	211 (94.2%)	421 (94.0%)	
>1 days	14 (6.3%)	13 (5.8%)	27 (6.0%)	
Reoperation in 30-days				.653^1^
No	222 (99.1%)	221 (98.7%)	443 (98.9%)	
Yes	2 (0.9%)	3 (1.3%)	5 (1.1%)	
Readmission in 30-days				.315^1^
No	223 (99.6%)	221 (98.7%)	444 (99.1%)	
Yes	1 (0.5%)	3 (1.3%)	4 (0.9%)	
Wound complication				.176^1^
Missing	0 (.0%)	1 (.4%)	1 (.2%)	
No	223 (99.6%)	219 (98.2%)	442 (98.7%)	
Yes	1 (0.5%)	4 (1.8%)	5 (1.1%)	
Venous thromboembolism				.317^1^
No	223 (99.6%)	224 (100.0%)	447 (99.8%)	
Yes	1 (.5%)	0 (.0%)	1 (.2%)	
Urinary tract infection				NA
No	224 (100.0%)	224 (100.0%)	448 (100.0%)	
Intra-op/post-op transfusion				.315^1^
No Complication	223 (99.6%)	221 (98.7%)	444 (99.1%)	
Transfusions/intra-op/post-op	1 (.5%)	3 (1.3%)	4 (.9%)	
Sepsis				0.156^1^
No	222 (99.1%)	224 (100.0%)	446 (99.6%)	
Yes	2 (.9%)	0 (.0%)	2 (.5%)	

∗Propensity score match for age, mFI-5, cardiac comorbidity, and “White” race.

1 Chi-Square.

2 Kruskal-Wallis.

## Discussion

Overall 30-day complication, reoperation, and readmission rates did not vary with resident presence. Operative time and patient length of stay were also similar across resident and no resident cohorts. In the 626 HA cases (35.8% with resident involvement) analyzed, our investigation found that, overall, resident involvement had no significant effect on 30-day HA outcomes.

This study used the ACS NSQIP database to identify potential surgical risk factors by analyzing patient demographic and comorbidity data from nearly 700 hospitals across the United States. Our unmatched results indicated that patients undergoing HA with a resident present are more likely to be younger, have a lower mFl-5 score, are less likely to be White, and are less likely to have a cardiac comorbidity (*P* < .05 for all). Together, these results indicate a generally healthier patient population.^37^ After cases were matched by propensity score to reduce bias, however, the only demographic variable that differed between cohorts was that patients assigned a resident were more likely to take corticosteroids for a chronic condition (*P* = .045). It has been postulated that surgical residents may be preferentially assigned to “teaching cases,” in which the procedures are considered riskier or more complex on account of the higher number of patient comorbidities in the demographic seeking care at academic hospitals.^9^^,^^24^ Although the findings of our matched analysis offer some support to these reports by suggesting that patients who undergo surgery with a resident present are less healthy, lower mFl-5 scores have been shown to be an effective predictor of less patient mortality and is relatively effective in predicting fewer postoperative complications.^37^ On the basis of these data, we are unable to substantiate the hypothesis that residents are selectively assigned to HA patients with medical histories that increase their risk of postoperative morbidity.

Regarding surgical outcomes, this study found that arthroscopies performed with a resident present did not have significantly longer operative times or higher complication rates in both the matched and unmatched analyses. Additionally, the complication and reoperation rates in each analysis outperformed the already low rates (4.1%-7.5% and 4.03%-6.3%, respectively) previously reported in the hip arthroscopy literature,^34^ thereby reinforcing prior claims about the safety of resident involvement in surgery. However, while the majority of studies agree that resident involvement does not significantly influence orthopedic outcomes, there is converging evidence to demonstrate that it does increase operative times. Although this study found a small difference in mean operative times between the resident and no resident cohorts, it was statistically, and likely clinically, insignificant. These results contradict our hypothesis by indicating that resident involvement was not a significant contributor to increased operative times in HA. Although these results are encouraging, surgeons should always be cognizant of any increase in operative time, as it is a well-known risk factor for surgical complications, including infection and traction-related neuropathy.

Resident education in surgery is an integral part of the graduate medical curriculum but concerns exist regarding patient perceptions of resident involvement in the OR and its influence on surgical outcomes.^19^^,^^38^ Although prior NSQIP studies have reported on the lack of negative impact of resident involvement in other areas of orthopedic surgery, the effects of resident involvement on the outcomes of hip arthroscopy have been long overlooked. This investigation successfully compared the outcomes of HA cases with and without resident involvement by using a large sample size from the ACS NSQIP database to perform a propensity score match analysis. Overall, the present study confirmed that complication rates in hip arthroscopy were low. Resident involvement did not increase the risk of adverse outcomes, readmission, reoperation, or length of stay, nor did it significantly increase operative times. Though surgical indications and techniques have evolved since 2012, these results are corroborated by the findings of previous orthopedic studies and help clarify the existing conflict in the literature regarding operative times by demonstrating that duration was not associated with resident involvement in hip arthroscopies. This study focuses ongoing efforts to elucidate the effects of resident involvement in surgery on the outcomes of HA. In doing so, it further defines its impact on the burgeoning cohort of hip patients by demonstrating that the presence of residents in the OR had no significant effect on short-term surgical outcomes.

### Limitations

The findings of this study are not without limitations, primarily due to the time range, length of follow-up (30 days), and completeness of the data available in the ACS NSQIP database. First, and most significantly, the database does not specify the amount of intraoperative resident involvement. As a result, whether a resident is performing, participating in, or simply observing a given procedure cannot be known and precludes this study from drawing any causative relationships between resident involvement and surgical outcomes. Second, the specialty and educational level of the surgeon(s) involved is not reported. Given the complexity of hip arthroscopy, resident and attending level of experience with a given procedure would invariably contribute to its outcomes. Third, the NSQIP database does not provide details about perioperative care. When we consider the multitude of orthopedic and nonorthopedic professionals involved in patient care, particularly in postoperative management, it is possible that these factors may influence surgical outcomes. Fourth is that the techniques used for each operation were not included with the CPT codes that were used to identify hip arthroscopies in the NSQIP database, making it impossible for us to control for any confounding factors related to varying surgical techniques.

Finally, and crucially, these findings only represent a sample, albeit a large one, of hip arthroscopies within a given database between 2006 and 2012, since that was the last year the NSQIP collected data on resident involvement. As a result, many cases were excluded from this analysis, and there is a large gap of time between the most recently collected data and the date of our retrospective query. This produces a potential risk of bias; however, it is worth noting that none of the medical comorbidities analyzed varied significantly between cases with and without resident data, indicating similar patient profiles between groups (Appendix 1). Not only have surgical indications and techniques developed over the past decade and the number of annual hip arthroscopies performed in the United States increased since 2012,^34^ but the NSQIP database is also restricted by the surgical settings that are included; for example, these results do not encompass independent surgical centers. Thus, the generalizability of these findings is limited accordingly and these conclusions about the impact of resident involvement should not be taken out of context. While this analysis has made valuable inroads for a long overlooked subject, the lack of more recent data on resident involvement in HA emphasizes a great need for their continued collection.

### Conclusions

Resident involvement in HA procedures was not a risk factor for 30-day complications between 2006 and 2012. Resident involvement did not increase the risk of adverse outcomes, readmission, reoperation, or length of stay, nor did it significantly increase operative times.

## Figures and Tables

**Appendix Table 1:** Hip Arthroscopy Demographics Bivariate Analysis—Resident Data Present Versus Missing

Variable	Resident Data Missing (*N* = 243)	Resident Data Present (*N* = 626)	Total (*N* = 869)	*P* Value
Sex				.077^1^
Female	161 (66.3%)	374 (59.7%)	535 (61.6%)	
Male	82 (33.7%)	252 (40.3%)	334 (38.4%)	
Age				**.038**^2^
*N*	243	626	869	
Mean (SD)	42.5 (14.4)	40.2 (14.0)	40.9 (14.1)	
Median	42.0	40.0	41.0	
Q1, Q3	31.0, 52.0	30.0, 48.0	30.0, 49.0	
mFI-5 Index				.549^2^
*N*	243	626	869	
Mean (SD)	.0 (.1)	.0 (.01)	.0 (.1)	
Median	.0	.0	.0	
Q1, Q3	.0, .0	.0, .0	.0, .0	
Race				**<.001**^1^
Asian	10 (4.1%)	5 (.8%)	15 (1.7%)	
Black or African American	7 (2.9%)	28 (4.5%)	35 (4.0%)	
Hispanic	11 (4.5%)	17 (2.7%)	28 (3.2 %)	
Other	24 (9.9%)	165 (26.4%)	189 (21.7%)	
White	191 (78.6%)	411 (65.7%)	602 (69.3%)	
Obese				.199^1^
No	168 (69.1%)	460 (73.5%)	628 (72.3%)	
Yes	75 (30.9%)	166 (26.5%)	241 (27.7%)	
ASA				.231^1^
<3	213 (87.7%)	566 (90.4%)	779 (89.6%)	
≥3	30 (12.3%)	60 (9.6%)	90 (10.4%)	
Diabetes				.897^1^
No	233 (95.9%)	599 (95.7%)	832 (95.7%)	
Yes	10 (4.1%)	27 (4.3%)	37 (4.3%)	
Dyspnea				.818^1^
Yes at moderate exertion	3 (1.2%)	9 (1.4%)	12 (1.4%)	
No	240 (98.8%)	617 (98.6%)	617 (98.6%)	
History of severe COPD				.084^1^
No	239 (98.4%)	623 (99.5%)	862 (99.2%)	
Yes	4 (1.6%)	3 (.5%)	7 (.8%)	
Cardiac comorbidity				.534^1^
No	204 (84.0%)	536 (85.6%)	740 (85.2%)	
Yes	39 (16.0%)	90 (14.4%)	129 (14.8%)	
Neurological comorbidity				.162^1^
No	243 (100.0%)	621 (99.2%)	864 (99.4%)	
Yes	0 (.0%)	5 (.8 %)	5 (.6%)	
Renal comorbidity				NA
No	243 (100.0%)	626 (100.0%)	869 (100.0%)	
Cumulative comorbidities				.286^1^
No	145 (59.7%)	398 (63.6%)	543 (62.5%)	
Yes	98 (40.3%)	228 (36.4 %)	326 (37.5%)	
History of Bleeding Disorders				.718^1^
No	240 (98.8%)	620 (99.0%)	860 (99.0%)	
Yes	3 (1.2%)	6 (1.0%)	9 (1.0%)	
In-patient/out-patient				.794^1^
In-patient	20 (8.2%)	55 (8.8%)	75 (8.6%)	
Out-patient	223 (91.8%)	571 (91.2%)	794 (91.4%)	
Functional Health Status Prior to Surgery				.098^1^
Independent	243 (100.0%)	619 (98.9%)	862 (99.2%)	
Partially dependent	0 (.0%)	6 (.1%)	6 (.7%)	
Totally dependent	0 (.0%)	1 (.2%)	1 (.1%)	
Current Smoker within One Year				.801^1^
No	199 (81.9%)	508 (81.2%)	707 (81.4%)	
Yes	44 (18.1%)	118 (18.8%)	162 (18.6%)	
Steroid Use for Chronic Condition				.193^1^
No	238 (97.9%)	620 (99.0%)	858 (98.7%)	
Yes	5 (2.1%)	6 (1.0%)	11 (1.3%)	

NOTE. Boldface indicates statistical significance (*P* < .05).

There were no significant differences in the patient comorbidities between the cases that were included in the analysis and those that were excluded. ASA, American Society of Anesthesiologist class; COPD, chronic obstructive pulmonary disease; mFl-5, 5-Factor Modified Frailty Index.

1 Chi-Square.

2 Kruskal-Wallis.

## Supplementary Data

ICMJE author disclosure forms
